# Developing a risk scoring system based on immune-related lncRNAs for patients with gastric cancer

**DOI:** 10.1042/BSR20202203

**Published:** 2021-01-06

**Authors:** Yuzhi Wang, Yu Zou, Yi Zhang, Chengwen Li

**Affiliations:** 1 Department of Clinical Laboratory, People’s Hospital of Deyang City, Deyang, China; 2 Department of Blood Transfusion, People’s Hospital of Deyang City, Deyang, China; 3 Department of Immunology, Southwest Medical University, Luzhou, China

**Keywords:** gastric cancer, immune grouping, lncRNAs, risk score

## Abstract

The immune system and the tumor interact closely during tumor development. Aberrantly expressed long non-coding RNAs (lncRNAs) may be potentially applied as diagnostic and prognostic markers for gastric cancer (GC). At present, the diagnosis and treatment of GC patients remain a formidable clinical challenge. The present study aimed to build a risk scoring system to improve the prognosis of GC patients. In the present study, ssGSEA was used to evaluate the infiltration of immune cells in GC tumor tissue samples, and the samples were split into a high immune cell infiltration group and a low immune cell infiltration group. About 1262 differentially expressed lncRNAs between the high immune cell infiltration group and the low immune cell infiltration group. About 3204 differentially expressed lncRNAs between GC tumor tissues and paracancerous tissues were identified. Then, 621 immune-related lncRNAs were screened using a Venn analysis based on the above results, and 85 prognostic lncRNAs were identified using a univariate Cox analysis. We constructed a prognostic signature using LASSO analysis and evaluated the predictive performance of the signature using ROC analysis. GO and KEGG enrichment analyses were performed on the lncRNAs using the R package, ‘clusterProfiler’. The TIMER online database was used to analyze correlations between the risk score and the abundances of the six types of immune cells. In conclusion, our study found that specific immune-related lncRNAs were clinically significant. These lncRNAs were used to construct a reliable prognostic signature and analyzed immune infiltrates, which may assist clinicians in developing individualized treatment strategies for GC patients.

## Introduction

Gastric cancer (GC) is the third-leading cause of cancer-related death and the fifth most common type of cancer worldwide [1]. It is a highly heterogeneous carcinoma due to its complicated molecular mechanism of carcinogenesis, such as genetic alterations, epigenetic changes, infection, and interaction with the microenvironment [2,3]. Despite the advancement of surgical resection, radiotherapy, and adjuvant chemotherapy techniques, the 5-year survival for GC is still very low in developing countries, and more than 90% of the GC patients are already at the advanced stages at diagnosis [4]. Overall, the prognosis of the disease is not optimistic. It is necessary to identify new biomarkers that would determine the progression of GC and explore the molecular mechanism of GC to reliably predict survival outcomes.

Current knowledge on the interactions between tumor and the immune system has laid the foundations for rationally guided stratification [5]. As reported by Wang et al., the interplay between different types of immune cells and GC cells is closely related to the occurrence, development, and prognosis of GC [6]. Moreover, long non-coding RNAs (lncRNAs) have been reported to be involved in the cross-talk between tumor cells and the tumor microenvironment, mainly composed of the extracellular matrix, stromal cells, and infiltrating immune cells [7–9]. The up-regulation of lncRNA CamK-A was observed in multiple human cancers, and it could regulate Ca^2+^-signaling-mediated tumor microenvironment remodeling, which includes macrophage recruitment, angiogenesis, and tumor progression [10]. LncRNAs are a class of non-coding RNAs with over 200 nucleotides in length that regulate gene expression at multiple levels from transcription to protein localization and stabilization [11,12]. Increasing evidence suggested that the dysregulation of lncRNAs play critical functional roles in the occurrence and development of GC [13–16]. For example, the up-regulation of lncRNA MIR4435-2HG in GC is linked to a late TNM stage and may promote GC metastasis by targeting desmoplakin to affect Wnt/β-catenin signaling [15]. Up-regulation of lncRNA BANCR decreased the apoptosis of GC cells by regulating NF-κB1 expression [17]. Down-regulation of lncRNA ncRuPAR [18] and MEG3 [19] in GC samples was associated with lymph node metastases, distant metastases, tumor size, and tumor invasion depth.

The fast-paced development of bioinformatics during recent times and the increasing availability of transcriptome data and clinical information have created conditions favorable for exploring the pathogenesis of gastric cancer. Personalized therapy has been projected to play a critical role in the prognosis of GC patients. The role of immune-related lncRNAs in GC remains mostly unknown. Therefore, the present study aimed to explore the role of immune-related lncRNAs to develop a risk scoring system for GC.

## Materials and methods

### Data acquisition

The RNA-sequencing data (fragments per kilobase of exon per million reads mapped (FPKM)) and corresponding clinical records of the GC patients were downloaded from the TCGA database (https://cancergenome.nih.gov/). These samples contained 375 GC tumor tissues and 32 paracancerous tissues.

### GC data processing and grouping

The gene markers of the immune cells were obtained from a previous study [20]. We analyzed 29 immune data sets based on these gene markers. Single-sample gene-set enrichment analysis (ssGSEA) is a unique GSEA method used to calculate separate enrichment scores for each sample and gene set. The activity or infiltration levels of the different immune cell types, immune-related functions, and immune-related pathways in the GC tissue samples were quantified using ssGSEA [21]. Then, unsupervised clustering was used to divide all GC tumor tissue samples into two groups based on the ssGSEA results using the R package, ‘hclust’. The first group was composed of samples with high immune cell infiltration (cluster 1), while the second group was composed of samples with low immune cell infiltration (cluster 2). Then, we validated the effectiveness of immune grouping in many ways. Based on the transcriptome expression profiles of the GC tumor tissues, the differentially expressed genes, Stromal Score Immune Score, ESTIMATE Score, and Tumor Purity were estimated using the ESTIMATE algorithm. The results are plotted and are shown in the clustering heat map and statistical map. Additionally, we applied the CIBERSORT deconvolution algorithm to precisely measure immune cell composition in the two subgroups.

### Screening for immune-related lncRNAs and prognostic lncRNAs

In the present study, lncRNAs with |logFC| > 1 and FDR < 0.05 between the two groups were identified as differentially expressed lncRNAs using the ‘edgeR’ package. Based on the criteria mentioned above, we identified differentially expressed lncRNAs between the high immune cell infiltration group and the low immune cell infiltration group. We also identified differentially expressed lncRNAs between the GC tissue group and paracancerous tissue group using the same method. Then, we extracted the immune-related lncRNAs based on the results of the two differentially expressed analyses mentioned above using a two-way Venn analysis. Next, the prognostic value of the immune-related lncRNAs was assessed through a univariate Cox proportional hazards analysis. The immune-related lncRNAs associated with patient prognosis (*P*-value < 0.05) were regarded as prognostic lncRNAs. The expression levels of the prognostic lncRNAs were significantly associated with the overall survival (OS) of the GC patients.

### Construction of a prognostic risk score model

To construct a stable prognostic risk score model, we randomly divided all data sets into two sets (training set: test set = 7:3). The least absolute shrinkage and selection operator (LASSO) is a method frequently used for data dimension reduction and indicators selection based on a generalized linear model [22]. Using the prognostic lncRNAs for GC, we screened lncRNAs with non-zero coefficients and established an optimal prognostic signature by combining LASSO regression analysis with 10-fold cross-validation in the training set. The risk score value based on the prognostic signature was calculated for each patient using the following formula: risk score = ∑ Coef_i_ * Expr_i_. GC patients were divided into a high-risk group and a low-risk group based on the median risk score. We constructed the same signatures using the test set and whole set to evaluate signature robustness. The Kaplan–Meier curves analysis was used to evaluate the survival differences among the high-risk and low-risk groups in each of the three sets. The receiver operating characteristic (ROC) curves of the whole set were drawn to appraise the performance of the risk score and parameters including age, grade, gender, T, M, N, and other previously published signatures. Additionally, we analyzed the risk score and some clinical features of the whole set using the univariate and multivariate Cox regression analyses to select independent prognostic factors.

### Functional enrichment analyses of the lncRNAs in the signature

The expression correlation analysis between the lncRNAs and mRNAs was used to identify the putative genes of lncRNAs in the signature. The absolute value of the Pearson correlation coefficient > 0.4 and a *P* value of < 0.0001 were set as the cutoff values. Gene Ontology (GO) and Kyoto Encyclopedia of Genes and Genomes (KEGG) enrichment analysis were performed using the R package, ‘clusterProfiler’.

### Associations between immune cell infiltration and risk score

The TIMER online database (https://cistrome.shinyapps.io/timer/) was analyzed, and the abundances of six types of infiltrating immune cells (B cells, CD4 T cells, CD8 T cells, neutrophils, macrophages, and dendritic cells) visualized. This analysis and visualization are ideal for detecting correlations between infiltrating immune cells and other factors. We downloaded the abundances of immune cells in GC patients and calculated the relationships between the risk score and infiltrating immune cells using Pearson correlation analysis.

## Results

### Construction and validation of GC groupings

**Figure 1:**
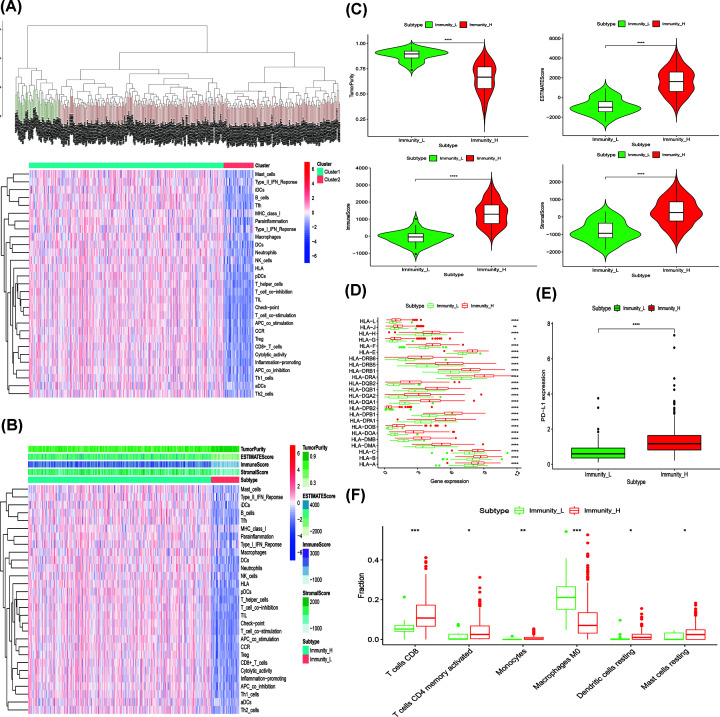
Construction and validation of gastric cancer groupings (**A**) Hierarchical clustering of 29 immune cells for 375 GC tumor tissues. (**B**) Using ESTIMATE algorithm, the Tumor Purity, ESTIMATE Score, Immune Score, and Stromal Score of each sample gene were shown together with the grouping information. (**C**) The box-plot showed that there was a statistical difference in Tumor Purity, ESTIMATE Score, Immune Score, and Stromal Score between the above two groups. (**D**) The expression of HLA family genes in high immune cell infiltration group were significantly higher than that in low immune cell infiltration group. (**E**) The expression of PD-L1 in high immune cell infiltration group were significantly higher than that in low immune cell infiltration group. (**F**) The statistical chart after using the CIBERSORT method showed the proportion difference of immune cell between the high immune cell infiltration group and the low immune cell infiltration group. ‘Immunity_H’ (Cluster1) represented the high immune cell infiltration group and ‘Immunity_L’ (Cluster2) represented the low immune cell infiltration group; *P<0.05, **P<0.01, ***P<0.001.

In the present study, we evaluated the infiltration of immune cells by analyzing the transcriptome of GC using the ssGSEA method and divided the GC tumor tissue samples into two groups using an unsupervised hierarchical clustering algorithm: the ‘Immunity-H’ group (sample size of high immune cell infiltration group = 325) and ‘Immunity-L’ group (sample size of low immune cell infiltration group = 50) (Figure 1A,B). The ESTIMATE method was used to uncover immune heterogeneity between the high immune cell infiltration group and the low immune cell infiltration group to validate the feasibility of the above immune grouping strategy. The results were used to draw statistical maps showing that the ‘Immunity-H’ group had a higher ESTIMATE Score, Immune Score and Stromal Score but lower Tumor Purity, compared with the ‘Immunity-L’ group (Figure 1C). We also validated the differences between the two groups by analyzing the gene expression level of the human leukocyte antigen (HLA) and PD-L1. The results suggested that the expression of the HLA family and PD-L1 in the ‘Immunity-H’ group were higher than that of the ‘Immunity-L’ group, respectively (*P*-value < 0.05) (Figure 1D,E). CIBERSORT analysis revealed that the proportion of most immune cells in the ‘Immunity-H’ group was higher than that in the ‘Immunity-L’ group. The results of the analysis, along with further details, are shown in Figure 1F. The above results together suggested that the immune grouping of GC was reasonable and adequate and was suitable for use in the subsequent analysis.

### Identification of differentially expressed immune-related lncRNAs

**Figure 2:**
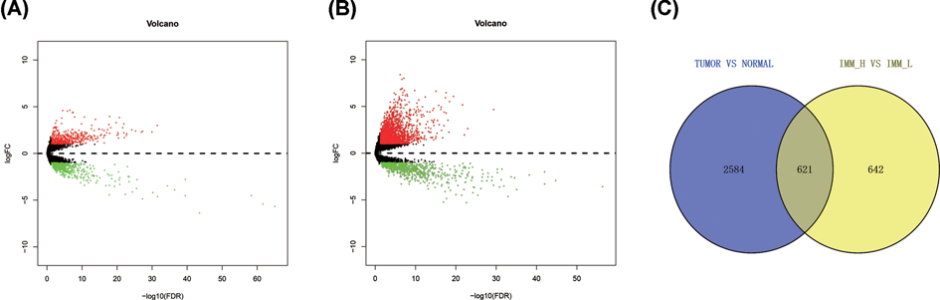
Identification of differentially expressed lncRNAs (**A**) Differentially expressed lncRNAs between the high immune cell infiltration group and the low immune cell infiltration group. (**B**) Differentially expressed lncRNAs between GC tumor tissue samples and paracancerous tissue samples. (**C**) Venn diagram showed the intersection of differentially expressed lncRNAs; lncRNA, long non-coding RNA.

About 1262 differentially expressed lncRNAs were identified between the high immune cell infiltration group and the low immune cell infiltration group, which included 514 up-regulated lncRNAs and 748 down-regulated lncRNAs (Figure 2A). Additionally, a total of 3204 differentially expressed lncRNAs were identified between GC tissues group and paracancerous tissues group, which included 2364 up-regulated lncRNAs and 840 down-regulated lncRNAs (Figure 2B). Using the intersection of these two results, we identified 621 lncRNAs as the differentially expressed immune-related lncRNAs (Figure 2C). The immune-related lncRNAs are associated with immunity and GC.

### Screening for prognostic lncRNAs

**Figure 3:**
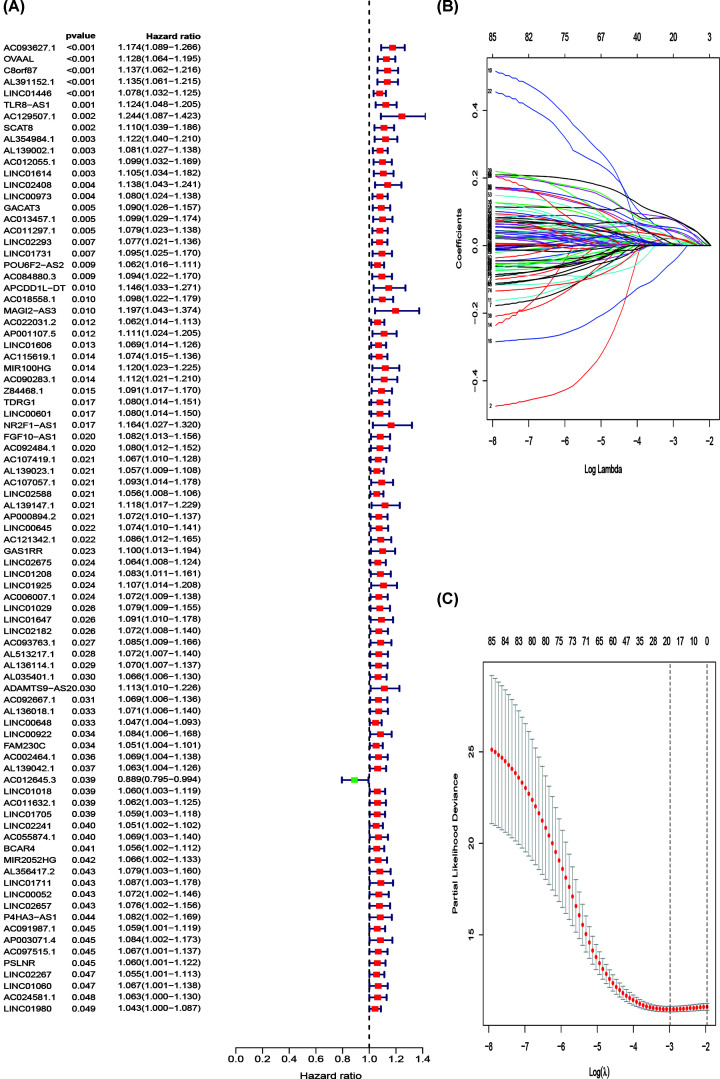
Construction of a prognostic risk score model (**A**) Forest plot of prognostic lncRNAs: the red dots represented prognostic lncRNAs with hazard ratios > 1 (*P*<0.05) and the green dots represented prognostic lncRNAs with hazard ratios < 1 (*P*<0.05). (**B**) The LASSO Cox analysis of the 85 prognostic lncRNAs. (**C**) Tuning parameter (lambda) selection in the LASSO model were determined by 10-fold cross-validation; lncRNA, long non-coding RNA.

Based on the findings presented above and clinical information corresponding with GC patients, we conducted a univariate Cox regression analysis to investigate the relationships between the 621 immune-related lncRNAs and patient prognosis. As a result, 85 immune-related lncRNAs were found to be significantly associated with OS (*P*-value < 0.05) and were defined as the prognostic lncRNAs in the training set (Figure 3A).

### Construction, verification, and evaluation of a prognostic signature

**Figure 4:**
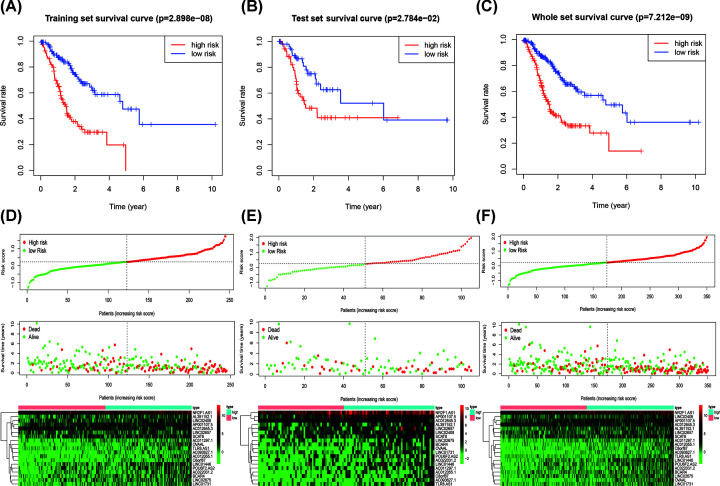
Verification of the prognostic signature Kaplan–Meier curve analysis of the high-risk and low-risk patients grouping by the prognostic signature in the training set (**A**), test set (**B**), and the whole set (**C**). Risk score distribution, survival status scatter plots, and expression patterns of risk genes for patients in the training set (**D**), test set (**E**), and the whole set (**F**).

**Figure 5:**
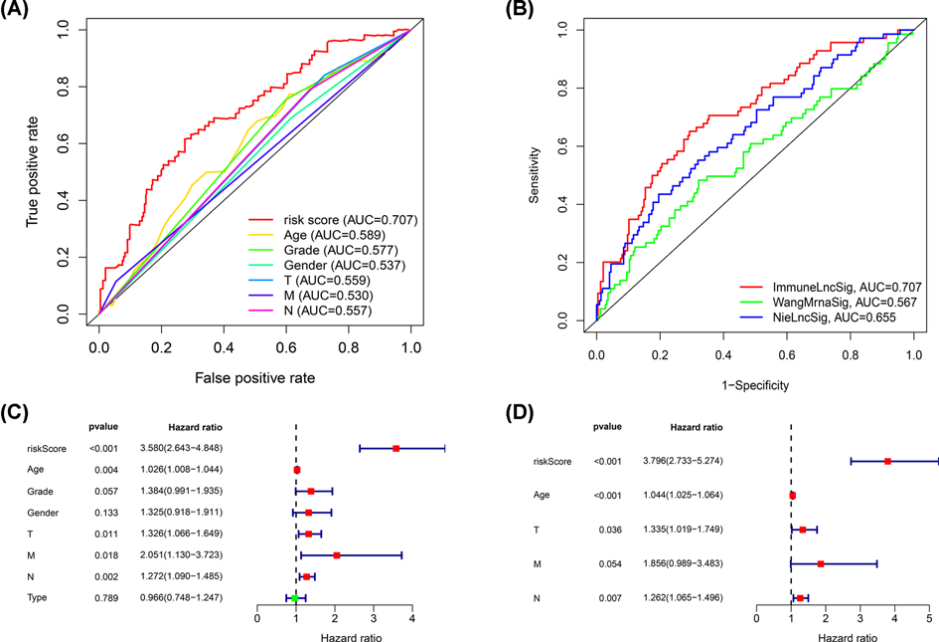
Evaluation of the prognostic signature (**A**) Receiver operating characteristic curve analyses in predicting the overall survival of patients for risk score, age, grade, gender, T, M, and N. (**B**) Receiver operating characteristic curve analyses in predicting the overall survival of patients for ImmuneLncSig, WangMrnaSig, and NieLncSig. (**C**) Univariate Cox regression analysis of risk score and clinical features including age, grade, gender, T, M, N, and type for the whole set. (**D**) Multivariate Cox regression analysis of risk score and clinical features including age, T, N, M, and risk score for the whole set.

**Figure 6:**
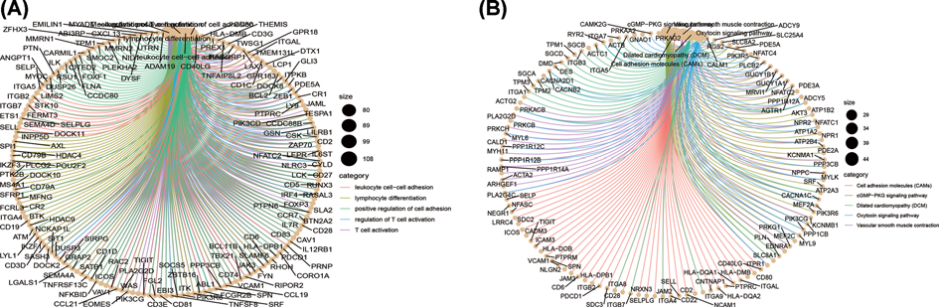
Functional enrichment analyses of the lncRNAs in the prognostic signature (**A**) The results of Gene Ontology analysis of the lncRNAs. (**B**) The results of Kyoto Encyclopedia of Genes and Genomes enrichment analysis of the lncRNAs; lncRNA, long non-coding RNA.

Personal clinical management requires the outcome of GC patients to be closely monitored. Therefore, we aimed to identify molecular biomarkers that could serve as a feasible prognostic signature. First, GC patients were randomly divided into a training set (*n*=245) and a test set (*n*=105). We applied LASSO Cox regression analysis with 10-fold cross-validation to determine the optimal values of the penalty parameter. Overall, we identified 19 core lncRNAs from the 85 prognostic lncRNAs and constructed a prognostic signature (Figure 3B,C). The prognostic signature was composed of the following lncRNAs: AP001107.5, AC012055.1, NR2F1.AS1, AC012645.3, BCAR4, POU6F2.AS2, AC022031.2, AL391152.1, OVAAL, LINC02408, LINC02675, AC093627.1, LINC01446, LINC02657, TLR8.AS1, C8orf87, LINC01731, AC011297.1, and SCAT8. Risk score = ∑ Coef_i_ * Expr_i_, where Coef is the coefficient obtained through the LASSO Cox regression, and Expr is the expression of the signature genes. The coefficients are summarized in Table 1. Then, patients were divided into a high-risk group and a low-risk group according to the median risk score. Significant differences between the two groups were shown by the Kaplan–Meier survival analysis in the training set, test set, and the whole set. The survival probabilities of the high-risk groups were significantly lower than that of the low-risk groups (Figure 4A–C). The distributions of the risk score were plotted along with the corresponding survival outcome (Figure 4D–F). ROC curves were plotted to evaluate the predictive power of clinical factors based on specificity and sensitivity. The area under the ROC (AUC) value for the prognostic signature was 0.707, which indicated moderate potential in using the prognostic signature for survival monitoring (Figure 5A). More importantly, we compared the signature in our study with previously reported lncRNA-based markers and found that the signature showed a superior predictive capability for gastric cancer (Figure 5B) [23,24]. Variables including age, grade, gender, T, M, N, type, and risk score were assessed using a univariate Cox regression analysis for the whole set (Figure 5C). Variables with a *P*-value < 0.05 of were further selected for further analysis using multivariate Cox regression analysis. We also identified independent predictors of patient survival outcome, including age, T, N, and risk score (Figure 5D). Besides, to understand the functions of the genes in the prognostic signature, we performed a pathway enrichment analysis on the genes. GO and KEGG analyses showed that the lncRNAs are involved in leukocyte cell–cell adhesion, lymphocyte differentiation, positive regulation of cell adhesion, regulation of T-cell activation, T-cell activation, cell adhesion molecules (CAMs), cGMP–PKG signaling pathway, dilated cardiomyopathy (DCM), oxytocin signaling pathway, and vascular smooth muscle contraction (Figure 6A,B).

**Table 1:** The core genes and their corresponding coefficients were summarized

Gene	Coef	Gene	Coef	Gene	Coef	Gene	Coef
AP001107.5	0.075	POU6F2.AS2	0.004	LINC02675	0.018	C8orf87	0.053
AC012055.1	0.021	AC022031.2	0.007	AC093627.1	0.075	LINC01731	0.005
NR2F1.AS1	0.024	AL391152.1	0.041	LINC01446	0.003	AC011297.1	0.023
AC012645.3	-0.045	OVAAL	0.079	LINC02657	0.003	SCAT8	0.045
BCAR4	0.025	LINC02408	0.045	TLR8.AS1	0.022	/	/

Coef, Coefficient.

### Correlations between the prognostic signature and infiltration of immune cell in GC patients

**Figure 7:**
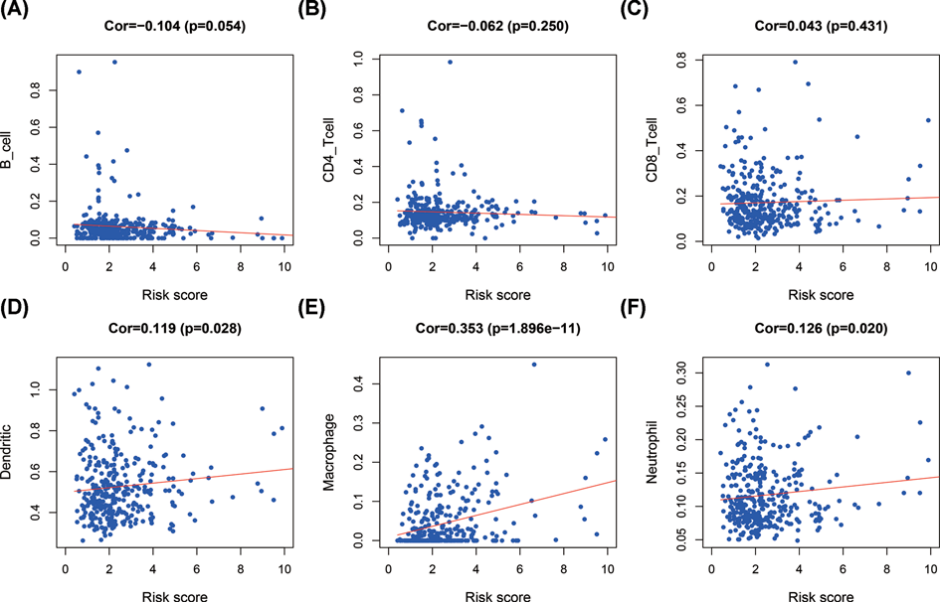
Correlations between the risk score and infiltration abundances of six types of immune cells B cells (**A**), CD4 T cells (**B**), CD8 T cells (**C**), dendritic cells (**D**), macrophages (**E**), and neutrophils (**F**).

Since the prognostic signature was relevant to tumor immunity, we analyzed the relationship between the risk score and abundances of six types of immune cells using data from the TIMER database (Figure 7A–F). As shown in the scatter diagrams, using a *P*-value of < 0.05 as the cut-off, the Pearson coefficients of DC, macrophages, and neutrophils with risk scores were 0.119, 0.353, and 0.126, respectively. The results showed that infiltration of DC, macrophages, and neutrophils was positively correlated with the risk score.

## Discussion

GC is a frequently encountered carcinoma that affects the upper digestive tract and results in high morbidity and mortality in China [25]. Tumor progression may be triggered by imbalances between the tumor and the host immune response [26]. In a previous report, patients with a highly infiltrated tumor microenvironment (TME) had more pre-existing immune reserves, suggesting that immune checkpoint inhibitor (ICI)-based monotherapy is likely to be useful for these patients. However, patients with a non-infiltrated TME always lack pre-existing immune reserves, indicating that ICIs alone are insufficient [5]. Each treatment regime may not be suitable for all patients owing to individual differences. Based on the importance of the immune landscape in the progression of cancer, we successfully divided GC tumor tissue samples into high immune cell infiltration and low immune cell infiltration groups by analyzing the overall immune cell infiltration characteristics. The powerful simplification of the immune grouping reflects a consequence of complex interactions between the tumor and the immune system. Recently, lncRNAs had become a hotspot for tumor research, and dysregulation of lncRNAs have been found to promoted the invasion and metastasis of GC [16,27]. Not only the immune system but lncRNAs also play essential roles during the development of GC. In our study, we constructed a risk score model based on immune-related lncRNAs. The survival curves show a high level of consistency among the three data sets, which indicates that GC patients with higher scores have a shorter duration of overall survival and a higher mortality rate than patients in the lower score group. The AUC value of 0.707 of the prognostic signature indicated that the risk score model had a more excellent relative prognostic value than T, M, N, gender, age and grade, and suggested good predictive performance. The multivariate Cox regression analysis showed that the prognostic signature functioned as an independent risk factor of GC.

The prognostic signature in our research consisted of 19 core immune-related lncRNAs and was found to be as reliable and valuable. Compared with single-gene biomarkers, signatures composed of multiple genes are generally more precise and more robust in predicting the outcomes of patients with cancer [28]. It can be used to determine the prognosis of patients with gastric cancer and assist clinical decision-making. Some lncRNAs in our prognostic signature had also been reported in some literatures. Similar to a previous study, our results illustrated in a forest plot showed that the expression of BCAR4 was significantly associated with the poor overall survival of GC patients [29]. Zhang et al. reported that LINC01446 was highly expressed in glioblastoma tissues and that LINC01446 deficiency inhibited glioblastoma cancer progression [30]. NR2F1-AS1 has been found to play a role in multiple types of cancer, including esophageal squamous cell carcinoma [31], colorectal cancer [32], and thyroid cancer [33]. As reported, TLR8-AS1 can be used as a diagnostic and prognostic marker and potential therapeutic target for ovarian cancer [34]. Most of the other lncRNAs in the signature have not been reported previously and need to be further explored.

In our study, GO and KEGG analyses showed that these lncRNAs were involved in leukocyte cell–cell adhesion, lymphocyte differentiation, positive regulation of cell adhesion, regulation of T-cell activation, T-cell activation, CAMs, cGMP–PKG signaling pathway, DCM, oxytocin signaling pathway, and vascular smooth muscle contraction. Previous studies have reported that cell adhesion molecules can alter cell adhesion and interactions between tumor cells, closely related to immune evasion [35]. cGMP/PKG signaling has been suggested to be closely associated with the tumor microenvironment. Activation of the cGMP/PKG pathway is generally accompanied by Wnt/β-catenin signaling that may help cancer cells evade surveillance from immune cells [36]. These results demonstrated that immune-related lncRNAs play significant roles in GC immunity. Additionally, positive correlations were observed between the risk score and the abundance of neutrophils, macrophages, and dendritic cells in GC, which indicates that the risk score may also serve as an immune status indicator. Therefore, the risk score can reflect the abundance of immune cells to rapidly adjust treatment plans.

For the first time, the immune system and lncRNAs, two critical factors that affect gastric cancer progression, have been combined to construct a prognosis signature for GC patients. Stratification of GC patients can help identify suitable therapeutic options for patients. For instance, a more aggressive treatment regime, stricter monitoring, and even novel clinical trials may be required for high-risk patients [37]. Personalized treatment options will not only improve the overall clinical outcome of patients but also magnify the benefits of clinical trials [37]. However, there were some shortcomings in the present study. First, our study was retrospective and the results should be further validated through prospective studies. Second, functional studies on the immune-related lncRNAs alone and in combination should be carried out to determine their suitability for clinical applications.

In summary, we screened for immune-related lncRNAs of clinical significance and used them to construct a reliable and valuable prognostic risk score model. The results of our study will enhance current knowledge base of tumor immunity and may offer a biological basis for the personalization of treatment options for GC patients.

## Data Availability

The data sets presented in this study can be found in online repositories (https://portal.gdc.cancer.gov/repository).

## Abbreviations

AUC: area under the ROC
CAM: cell adhesion molecule
DCM: dilated cardiomyopathy
GC: gastric cancer
HLA: human leukocyte antigen
ICI: immune checkpoint inhibitor
lncRNA: long non-coding RNA
OS: overall survival
TME: tumor microenvironment
